# Decoding uterine (dys)function in fibroids through multimodal assessment of functional determinants: a systematic review and meta-analysis

**DOI:** 10.1093/hropen/hoaf060

**Published:** 2025-09-18

**Authors:** Noemi Salmeri, Edgardo Somigliana, Alexander Fiore, Davide Marinello, Benedetta Maizza, Francesca Filippi, Paola Viganò, Fabio Parazzini

**Affiliations:** Department of Clinical Sciences and Community Health, Università degli Studi, Milan, Italy; Infertility Unit, Fondazione IRCCS Ca’ Granda Ospedale Maggiore Policlinico, Milan, Italy; Department of Clinical Sciences and Community Health, Università degli Studi, Milan, Italy; Infertility Unit, Fondazione IRCCS Ca’ Granda Ospedale Maggiore Policlinico, Milan, Italy; Infertility Unit, Fondazione IRCCS Ca’ Granda Ospedale Maggiore Policlinico, Milan, Italy; Infertility Unit, Fondazione IRCCS Ca’ Granda Ospedale Maggiore Policlinico, Milan, Italy; Department of Biomedical Sciences, Humanitas University, Pieve Emanuele, Italy; Infertility Unit, Fondazione IRCCS Ca’ Granda Ospedale Maggiore Policlinico, Milan, Italy; Infertility Unit, Fondazione IRCCS Ca’ Granda Ospedale Maggiore Policlinico, Milan, Italy; Department of Clinical Sciences and Community Health, Università degli Studi, Milan, Italy

**Keywords:** uterine fibroids, uterine vascularization, uterine arteries, elastography, uterine peristalsis

## Abstract

**Study Question:**

Is there a difference in uterine functional determinants between women with fibroids and women without myometrial pathology?

**Summary Answer:**

Women with uterine fibroids consistently exhibit altered uterine functional determinants compared to controls, characterized by increased perfusion, elevated stiffness, and impaired contractility.

**What is Known Already:**

The functional determinants of the non-pregnant uterus remain largely unexplored and underreported. Uterine fibroids, as a well-defined morphological myometrial pathology, offer a unique model for understanding uterine functionality.

**Study Design, Size, Duration:**

This systematic review and meta-analysis included original articles published in English and indexed in PubMed, Embase, and Scopus databases up to 20 December 2024. The search strategy combined terms related to uterine fibroids with those describing uterine functional parameters (e.g. uterine vascularity, stiffness, and contractility), together with diagnostic methods (including Doppler ultrasound, elastography, and magnetic resonance imaging).

**Participants/Materials, Setting, Methods:**

Observational studies evaluating quantitative uterine functional determinants in non-pregnant women with fibroids and controls without myometrial pathology were selected using predefined Population, Intervention (Investigated measure), Comparator, Outcome(s), Study type (PICOS) criteria. Outcomes included quantitative measures of uterine functionality such as vascularization (uterine artery Doppler indices), stiffness (elastography parameters), and contractility (peristalsis parameters). Study quality was evaluated using the Newcastle–Ottawa Scale. Pooled estimates for continuous outcomes were calculated using random-effects models, expressed as mean difference (MD) with 95% CIs. Subgroup analyses addressed potential confounders, including menopausal status, hormonal therapy use, and symptom severity.

**Main Results and the Role of Chance:**

Fourteen studies met the inclusion criteria: seven on vascularization (n = 961), five on stiffness (n = 342), and two on contractility (n = 62). The uterine artery pulsatility index was significantly lower in women with fibroids compared to controls (MD −0.63, 95% CI −0.91 to −0.36; *I*^2^ = 91.98%), with greater reductions observed in premenopausal, non-hormonally treated, and symptomatic women. The resistance index also decreased (−0.09, 95% CI −0.15 to −0.03; *I*^2^ = 95.86%), showing similar patterns across subgroups. Time-averaged maximum velocity was higher in the fibroid group (+18.46, 95% CI +5.54 to +31.37; *I*^2^ = 93.64%), particularly in premenopausal and symptomatic cases. Elastography showed increased myometrial stiffness in uterine fibroids compared to controls, with a higher elastic modulus (+35.58 kPa, 95% CI +24.94 to +46.22; *I*^2^ = 0%) and shear wave velocity (+1.14 m/s, 95% CI +0.62 to +1.65; *I*^2^ = 0%). Limited evidence pointed to reduced peristaltic activity and altered contraction patterns in symptomatic fibroids.

**Limitations, Reasons for Caution:**

The relatively small study population and high heterogeneity of estimates warrant cautious interpretation, although findings were consistent across multiple uterine functional determinants.

**Wider Implications of the Findings:**

Women with uterine fibroids consistently exhibit altered uterine functional determinants compared to controls without myometrial pathology, highlighting how structural abnormalities parallel functional changes. Leveraging fibroids as a model, integrating structural imaging with functional assessment through advanced multimodal approaches may deepen our understanding of uterine diseases, ultimately enhancing treatment and patient care.

**Study Funding/Competing Interest(s):**

This study was partially funded by the Italian Ministry of Health—Current research IRCCS. The funding source had no role in the study design; in the collection, analysis, or interpretation of data; in the writing of the report; or in the decision to submit the article for publication. E.S. reports payments from Ferring, Theramex, and IBSA for research grants and honoraria from IBSA, Gedeon-Richter, and Sandoz for lectures. He serves as Editor-in-Chief of *Human Reproduction Open*. P.V. has received honoraria as Co-Editor in Chief of *Journal of Endometriosis and Uterine Disorders*. The remaining authors have no conflicts of interest to disclose.

**Registration Number:**

PROSPERO ID: CRD42024619633—registered on 10 December 2024.

WHAT DOES THIS MEAN FOR PATIENTS? The uterus is a vital organ that not only supports pregnancy but also plays an important role in a woman’s overall health. However, how the uterus functions outside of pregnancy is still not fully understood. One of the most common conditions affecting the uterus is fibroids—benign growths in the uterine wall that can cause heavy periods, pelvic pain, and fertility problems. This systematic review explored how fibroids affect uterine function. We found that women with fibroids tend to have increased blood flow, stiffer uterine tissue, and weaker muscle contractions compared to women without fibroids. These differences were more pronounced in women who had not gone through menopause, those not using hormone therapy, and those with more severe symptoms. These findings suggest that fibroids not only change the shape of the uterus but also affect how it functions. Understanding these changes could help doctors develop better strategies to manage symptoms and improve care for women with fibroids, and possibly those with other uterine conditions.

## Introduction

At the heart of female reproductive health lies the uterus—a dynamic organ whose role extends far beyond gestation. Formed during intrauterine life, the uterus becomes functionally active at puberty (Habiba *et al.*, 2021). Cyclical hormonal changes prepare the uterus for embryo implantation or trigger menstruation through shedding of the lining when implantation does not occur, primarily driven by declining progesterone levels (Pavlicev and Norwitz, 2018).

Historically, research on uterine function has focused predominantly on the endometrium. However, significant intra- and inter-patient variability across menstrual cycles has been reported (Evans *et al.*, 2016; Critchley *et al.*, 2020).

In contrast, the myometrium—the muscular layer of the uterus—has been largely underexplored, with functional determinants often underreported, inconsistently measured, and poorly understood. Nevertheless, biological evidence underscores its crucial roles in reproductive outcomes and menstrual health (Bulun, 2013). Dysregulated uterine contractions leading to transient myometrial ischemia have been reported during menstrual pain, suggesting a complex interaction between uterine perfusion, muscle activity, and dysmenorrhea (Iacovides *et al.*, 2015; Cockrum *et al.*, 2024). Moreover, abnormal uterine peristalsis has been linked to retrograde menstruation and endometriosis development (Leyendecker *et al.*, 2004; Salmeri *et al.*, 2024) and may also adversely affect embryo placement and implantation success during ART (Fanchin *et al.*, 2001).

Although non-invasive assessment of myometrial function remains challenging, recent advances in diagnostic technologies are opening new avenues for investigation. Uterine artery Doppler ultrasound represents a validated tool for the indirect evaluation of uterine perfusion at the upstream vascular level (Fujino *et al.*, 1993; Hsieh *et al.*, 2000). Ultrasound-based elastography enables the quantitative assessment of myometrial stiffness by measuring tissue elasticity, providing a non-invasive window into the biomechanical properties of the uterine wall (Stoelinga *et al.*, 2014). Furthermore, innovations in dynamic imaging modalities and electrophysiology have significantly enhanced the ability to visualize and quantify uterine peristalsis *in vivo* (Kunz and Leyendecker, 2002; Fujiwara *et al.*, 2004; Wang *et al.*, 2023).

Unlocking the full clinical and physiological potential of these diagnostic tools requires a multidimensional approach to capture the complexity of biological processes. This, in turn, calls for a reliable and well-characterized pathological model. Uterine fibroids, characterized by distinctive ultrasonographic features and high diagnostic reliability (Van den Bosch *et al.*, 2015), serve as an ideal model for investigating how structural alterations of the myometrium may affect uterine function.

This systematic review and meta-analysis aims to integrate available evidence on key functional determinants of uterine vascularization, stiffness (assessed via elastography), and contractility in women with fibroids compared to controls, to deepen our understanding of structure–function relationships in uterine pathology.

## Methods

The meta-analysis protocol was registered on PROSPERO (CRD42024619633). The findings were reported following the Preferred Reporting Items for Systematic Reviews and Meta-Analyses (PRISMA) and the Meta-analysis of Observational Studies in Epidemiology (MOOSE) guidelines (Stroup *et al.*, 2000; Page *et al.*, 2021).

### Search strategy

Two reviewers (N.S. and B.M.) searched the PubMed, Embase, and Scopus databases from study inception up to 20 December 2024. The search strategy included a combination of Medical Subject Headings (MeSH) terms and equivalent keywords (Supplementary File S1). Only studies published in peer-reviewed journals with full-text availability in English were considered.

### Eligibility criteria

Inclusion criteria were defined using the Population, Intervention (Investigated measure), Comparator, Outcome(s), Study type (PICOS) framework as follows (Huang *et al.*, 2006):

1. Population: non-pregnant women with uterine fibroids. Accepted diagnostic methods included: (i) surgical visualization and histological confirmation; (ii) imaging techniques such as transvaginal ultrasound (TVUS), transabdominal ultrasound (TAUS), and/or magnetic resonance imaging (MRI). Diagnoses based solely on medical records or patient self-reporting were not considered eligible.
2. Intervention (Investigated measure): surrogate variables for the *in vivo* quantitative assessment of uterine functionality in non-pregnant women, including uterine vascularization, stiffness, and contractility.
3. Comparator: women without uterine fibroids. Studies including controls with known uterine conditions were excluded, unless stratified data were available or confounding factors appropriately addressed.
4. Outcomes: pooled mean differences between cases and controls in: (i) uterine vascularization, assessed by Doppler parameters of the uterine arteries (UtAs, average of left and right), including pulsatility index (PI), resistance index (RI), time-averaged maximum velocity (TAMX), and peak systolic velocity (PSV); (ii) uterine stiffness, measured by shear wave elastography (SWE) and/or strain elastography (SE), using variables such as elastic modulus, shear wave velocity (SWV), strain ratio (SR), or elasticity score; (iii) uterine contractility, evaluated by the presence of peristaltic waves, and the frequency and direction of uterine contractions.
5. Study type: only observational studies. Non-original articles (e.g. reviews, editorials), *in vitro* or animal studies, and conference abstracts without full-text data were excluded.

### Study selection and data extraction

Records were independently screened by two reviewers (B.M. and D.M.), first by title and abstract, followed by a full-text review based on eligibility criteria. Any discrepancies were resolved by consulting a third reviewer (N.S.). A full list of data abstracted and tabulated is reported in Supplementary File S2.

### Risk of bias assessment

Risk of bias assessment was conducted independently by two reviewers (B.M. and D.M.), using the Newcastle–Ottawa Scale (NOS) quality appraisal checklist (Wells *et al.*, 2021), with an adapted version for cross-sectional studies (Herzog *et al.*, 2013). Disagreements were resolved by discussion with a third reviewer (A.F.).

### Certainty of the evidence assessment

The certainty of the evidence was graded according to the Grading of Recommendations Assessment, Development, and Evaluation (GRADE) guidelines as high, moderate, low, or very low (Guyatt *et al.*, 2008).

### Data analysis

For studies that reported means and SEMs rather than SDs, SDs were calculated using standard statistical formulas as recommended in the Cochrane Handbook for Systematic Reviews of Interventions (Higgins *et al.*, 2023). For studies presenting medians along with interquartile ranges or ranges, means and SDs were estimated using methods described by Wan *et al.* (2014) and Luo *et al.* (2018). In studies evaluating uterine vascularization that provided separate data for right and left UtAs without reporting significant differences between sides, the average of the bilateral measurements was calculated and utilized for the analysis.

Pooled estimates were calculated using random-effects models (DerSimonian and Laird, 1986). Statistical heterogeneity was assessed using the *I*^2^ statistics, according to the Cochrane Handbook for Systematic Reviews of Interventions (Cochrane, 2023): 0–40% (not important), 30–60% (moderate), 50–90% (substantial), and 75–100% (considerable). Sensitivity analysis was performed to assess the effect of individual studies on the pooled estimate (leave-one-out meta-analysis). Subgroup analyses were conducted in cases of high heterogeneity to assess the impact of confounders. Mediators were chosen based on clinical relevance and data availability, requiring at least two studies per subgroup. Pre-planned subgroup analyses included ongoing hormonal treatment (HT), clinical presentation (symptoms and/or infertility), menopausal status, and menstrual cycle phase (in premenopausal women). Publication bias was assessed using Egger’s and Begg’s tests for small-study effects (Begg and Mazumdar, 1994; Egger *et al.*, 1997).

For study outcomes where a meta-analysis was based on only two studies, we applied alternative synthesis methods as recommended by the Cochrane Handbook for Systematic Reviews of Interventions (McKenzie and Brennan, 2024). Specifically, we used vote counting based on the direction of effect, classifying each estimate as positive (higher values in fibroids vs controls) or negative (lower values). The pooled proportion of positive effects was calculated as p^+^ = u^+^/n, where u^+^ is the number of positive effects and n the total number of studies. To determine whether the observed proportions differed from chance, two-sided binomial probability tests (sign tests) were applied under the conservative null hypothesis of equal probability (*P* expected = 0.5), and 95% CIs were calculated using the Wilson method (DasGupta *et al.*, 2001).

Analyses were performed using STATA software (Version 18.5, StataNow/SE; Stata Corp LLC, 2024, College Station, TX, USA).

## Results

**Figure 1.**
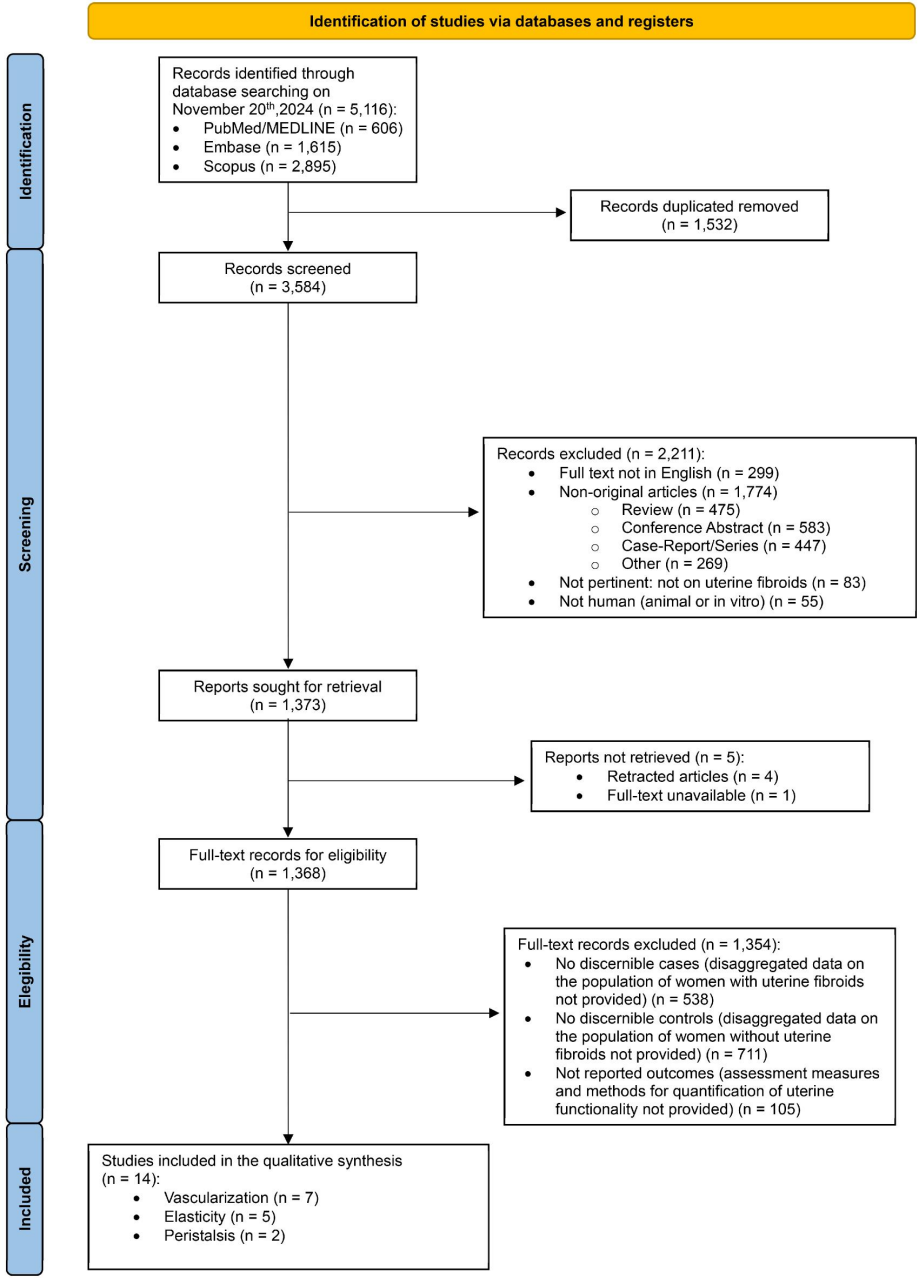
PRISMA 2020 flow diagram.

Of the 5116 records identified, 1532 duplicates were removed, leaving 3584 records for screening. Among these, 1368 full-text articles were assessed for eligibility, and 14 studies met the inclusion criteria. Specifically, seven studies evaluated uterine vascularization through UtA Doppler parameters (Kurjak *et al.*, 1992; Sladkevicius *et al.*, 1995, 1996; Alataş *et al.*, 1997; Yu Ng *et al.*, 2005; Samani *et al.*, 2012; Idowu *et al.*, 2017), five studies investigated uterine stiffness using elastography techniques (Zhang *et al.*, 2019; Görgülü and Okçu, 2021; Pongpunprut *et al.*, 2022; Săsăran *et al.*, 2022; Kaya Narçiçeği *et al.*, 2023), and two studies assessed uterine contractility (Orisaka *et al.*, 2007; Kido *et al.*, 2014). None investigated more than one aspect. The study selection process is summarized in the PRISMA flow diagram (Fig. 1). The reasons for exclusion from the quantitative synthesis are reported in Supplementary Table S1.

The risk of bias for all the included studies, according to the NOS, is reported in Supplementary Table S2.

### Uterine vascularization

A summary of the main characteristics of studies comparing uterine vascularization in women with fibroids and controls is reported in Table 1 and Supplementary Table S3.

**Table 1.** Baseline characteristics of included studies.

First author, year	Type of study	Sample size: total (cases/controls)	**Study population: (1) age** ^a^ **(2) reproductive history (3) hormonal and/or menopausal status**	Definition of cases (fibroids)	Uterine fibroids: (1) characteristics (2) symptoms	Definition of controls
**Uterine vascularization (n = 7 studies)**						
Alataş *et al.*, 1997	Case–control	160 (100/60)	(1) Cases: 42.8 ± 6.7; controls: 39.2 ± 10.4 (2) NR (3) All pre-menopausal; no HT	US detection and surgical/histological confirmation	(1) NR (2) NR	Healthy women attending annual gynecological checkups
Idowu *et al.*, 2017	Case–control	280 (140/140)	(1) Cases: 37.9 ± 7.4; controls: 30.5 ± 8.3 (2) Nulliparous: ∼60% of population (no difference cases vs. controls); excluded women with childbirth <1 year (3) All pre-menopausal	US detection	(1) NR (2) ∼86% symptomatic (menorrhagia, pain, abdominal swelling); ∼10% were recurrence after myomectomy	Women with leiomyoma-free uteri and normal endometrial thickness by US
Kurjak *et al.*, 1992 ^b^	Case–control	131 (71/60)	(1) Cases: 39 (26–58); controls: 33 (NR) (2) NR (3) All pre-menopausal; no HT	US detection, palpable at the gynecologic examination	(1) NR (2) NR	Healthy volunteers (negative US)
Yu Ng *et al.*, 2005	Case–control	100 (50/50)	(1) Cases: 36 (27–40); controls: 36 (27–40) (2) 80% with primary infertility (no difference cases vs. controls) (3) All pre-menopausal; all undergoing IVF-ET for specified causes of infertility (no difference cases vs. controls)	US detection	(1) Excluded pedunculated subserosal and fibroids causing endometrial cavity distortion (2) Infertility; none with history of myomectomy	Women with infertility undergoing IVF-ET (negative US)
Samani *et al.*, 2012	Case–control	100 (50/50)	(1) NR (2) NR (3) No HT	US detection	(1) Excluded submucosal, subserosal, cornual, and degenerated fibroids (2) NR	Women without uterine fibroids (by US), surgical history, or comorbidities
Sladkevicius *et al.*, 1995	Case–control	144 (27/117)	(1) Total cohort: 54 (45–64)^c^ (2) NR (3) All post-menopausal; no current or previous HT	US detection	(1) NR (2) NR	Asymptomatic women undergoing cervical cancer screening; negative exam, no previous pelvic surgery
Sladkevicius *et al.*, 1996 ^d^	Case–control	46 (28/18)	(1) Cases: 45 (30–53); controls: 39 (29–45) (2) NR (3) All pre-menopausal; no HT	US detection and surgical/histological confirmation	(1) NR (2) All symptomatic	Asymptomatic healthy volunteers (negative US)
**Uterine stiffness (n = 5 studies)**						
Görgülü and Okçu, 2021	Case–control	138 (98/40)	(1) Cases: 45.8 ± 7.8; controls: 45.4 ± 7.1 (2) NR (3) All pre-menopausal; no GnRH-agonist use during the last year in controls	US/MRI detection and surgical/histological confirmation	(1) NR (2) All with treatment-resistant menometrorrhagia or chronic pelvic pain	Healthy volunteers without uterine lesions (by US), pelvic infections, suspicion of malignancy
Kaya Narçiçeği *et al.*, 2023 ^e^	Cohort	56 (12/28)	(1) Cases: 47 (35–66); controls: 48 (39–76) (2) Parity: range 0–9 (no difference cases vs. controls) (3) Post-menopausal: ∼55% of population; no HT	Mapped by US prior to surgery and histologically confirmed post-surgery	(1) NR (2) NR	Normal myometrium mapped by US prior to surgery and histologically confirmed post-surgery
Pongpunprut *et al.*, 2022 ^e^	Cross-sectional	50 (25/25)	(1) Cases: 46.9 ± 16.1; controls: 62.5 ± 15.5 (2) Multiparity: 80% cases vs. 64% controls (3) Pre-menopausal: 76% cases vs. 16% controls; heterogeneous endometrial pathology (8% of cases on progestins)	Mapped by US and histologically confirmed post-surgery	(1) NR (2) NR	Normal myometrium mapped by US and histologically confirmed post-surgery
Săsăran *et al.*, 2022 ^f^	Case–control	63 (17/46)	(1) Cases: 47.1 ± 4.1; controls: 37.8 ± 7.8 (2) Previous CS: 4.76% cases vs. 19.04% controls (3) All pre-menopausal; no previous or present HT (GnRh agonist, COCs, IUD, implants)	All with concomitant uterine fibroids and adenomyosis (surgical/histological confirmation)	(1) NR (2) All symptomatic (treatment-resistant menometrorrhagia, dysmenorrhea, chronic pelvic pain or symptoms associated with pelvic compression)	Healthy women attending regular check-ups, with no history of malignancy or uterine infection
Zhang *et al.*, 2019	Cohort	34 (13^g^/15)	(1) Total cohort: 36.8 (22–52)^c^ (2) NR (3) All pre-menopausal	US/MRI detection (with or without concomitant adenomyosis)	(1) NR (2) All with pelvic pain and/or AUB	Women referred for imaging due to pelvic pain and/or AUB, with normal myometrium on MRI
**Uterine contractility (n = 2 studies)**						
Kido *et al.*, 2014	Case–control	40 (20/20)	(1) Cases: 45.5 ± 3.7; controls: 33.3 (19–46) (2) NR (3) All pre-menopausal; no HT	US/MRI detection and treatment by HIFU ablation	(1) Localization: intramural (n = 8), submucosal (n = 10), intracavitary (n = 1), subserosal (n = 1) (2) All symptomatic for pain and AUB	Healthy female staff volunteers (normal myometrium on MRI)
Orisaka *et al.*, 2007	Case–control (pilot)	22 (19/3)	(1) Cases: 34.8 (24–42); controls: 32 (28–36) (2) NR (3) All pre-menopausal; no HT	US/MRI detection and normal menstrual cycles	(1) Localization: intramural (n = 15), subserosal (n = 2), submucosal (n = 2) (2) 36.8% with hypermenorrhea	Healthy volunteers (normal myometrium on MRI and normal menstrual history)

HT, hormonal treatment; IVF-ET, IVF and embryo transfer; NR, not reported; US, ultrasound. AUB, abnormal uterine bleeding; COCs, combined oral contraceptives; CS, cesarean section; IUD, intrauterine device; HIFU, high intensity focused ultrasound.

a Age is reported as mean ± SD or median (interquartile range).

b The study presented disaggregated outcome data for vascularized and avascularized fibroids compared with the same control cohort. Given the larger sample size in the vascularized fibroid group (N = 71 vs N = 30), and to avoid data overlap, only data related to vascularized fibroids were included in the analysis.

c Disaggregated data for cases and controls were not reported.

d The study presented disaggregated data for pre- and post-menopausal women compared to the same control cohort. To avoid data overlap with the previous publication by the same authors, only data from pre-menopausal women were retrieved.

e Included women undergoing hysterectomy who underwent myometrial elastography the day before surgery. Marked areas were classified as normal, adenomyosis, or uterine fibroids based on histological analysis. Adenomyosis was excluded from comparisons.

f Excluded from quantitative synthesis.

g One case with concomitant adenomyosis.

#### UtA PI

**Figure 2.**
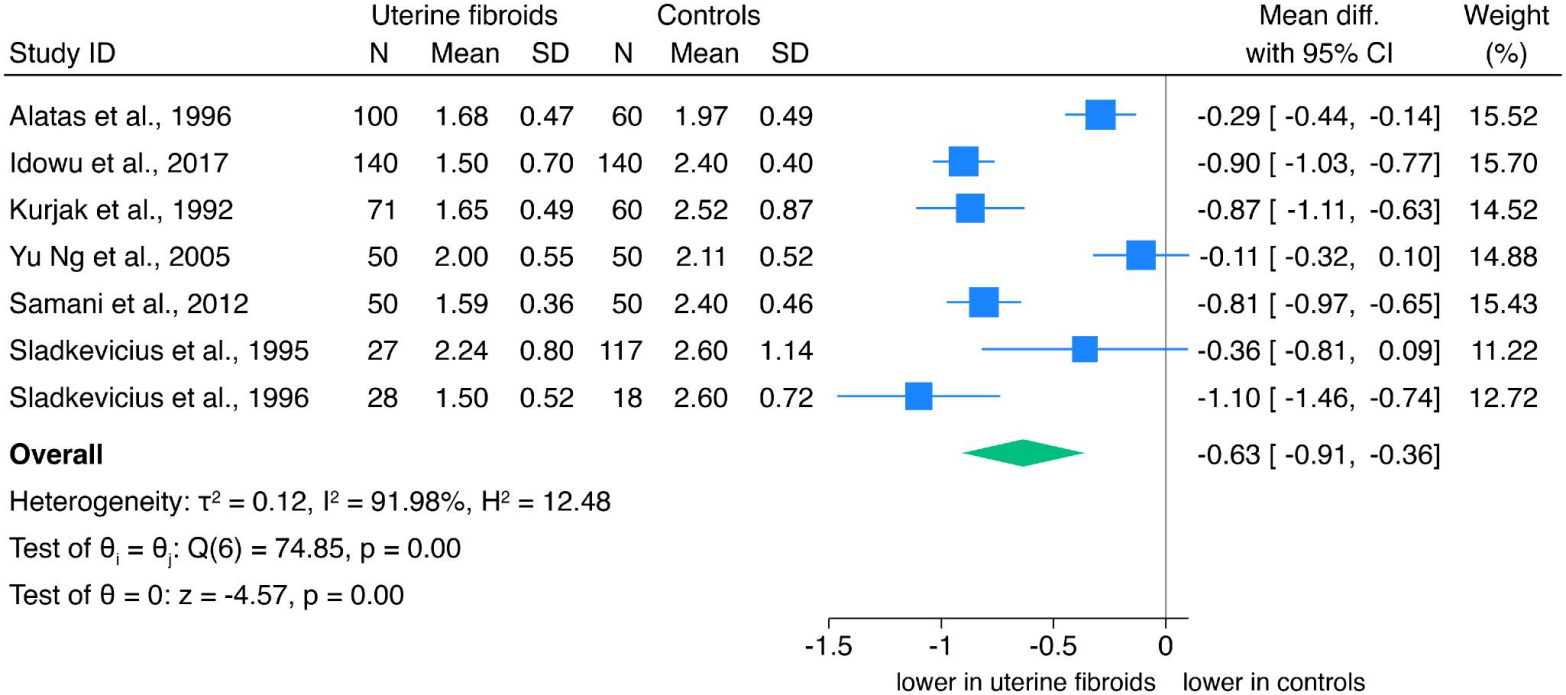
Forest plot of the meta-analysis on uterine artery pulsatility index (UtA PI) in women with uterine fibroids vs controls.

Seven studies (n = 961 women) examined UtA PI. Overall, UtA PI was significantly lower in women with fibroids (mean difference: −0.63; 95% CI, −0.91 to −0.36; *I*^2^ = 91.98%), with no publication bias (Egger’s: *z* = −0.04, *P* = 0.97; Begg’s: *z* = 0.30, *P* = 0.76) (Fig. 2). Sensitivity analysis confirmed the robustness of the estimate (Supplementary Fig. S1). According to GRADE, the certainty of the evidence was low (Supplementary Table S4).

**Figure 3.**
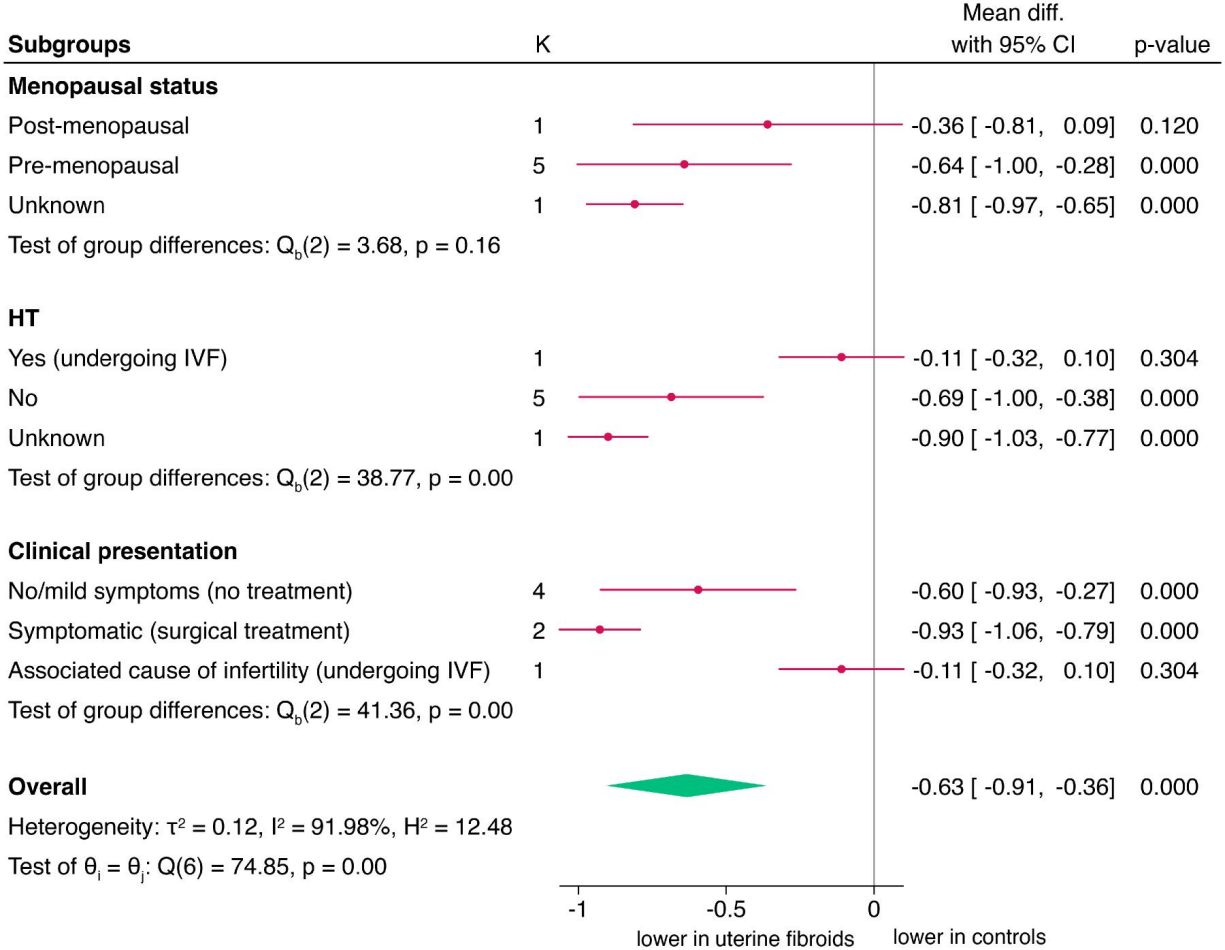
**Sub-group analysis of uterine artery pulsatility index (UtA PI) in women with uterine fibroids vs controls, according to menopausal status, hormonal treatment, and clinical presentation.** HT, hormonal treatment.

Subgroup analyses are reported in Fig. 3. Greater UtA PI reduction was observed in pre-menopausal women (pooled mean: −0.64; 95% CI, −1.00 to −0.28; n = 5 studies), but no significant difference in post-menopausal women. Women not using HT had lower UtA PI (pooled mean: −0.69; 95% CI, −1.00 to −0.38; n = 5 studies), whereas the only study on women using HT showed no difference (test of group differences: *P* < 0.01). Lower UtA PI were found in women with severe symptoms (pooled mean: −0.93; 95% CI, −1.06 to −0.79; n = 2 studies) compared to mild/asymptomatic women (pooled mean: −0.60; 95% CI, −0.93 to −0.27; n = 4 studies), with no difference reported in the study addressing infertile women undergoing IVF.

Sensitivity analysis on studies controlling for menstrual cycle phase (follicular) reported a pooled mean UtA PI of −0.57 (95% CI, −0.97 to −0.16; *I*^2^ = 87.64%; n = 2 studies) (Supplementary Fig. S2).

#### UtA RI

Five studies (n = 771 women) examined UtA RI, finding slightly lower values in women with fibroids (pooled mean: −0.09; 95% CI, −0.15 to −0.03; *I*^2^ = 95.86%) with no publication bias (Egger’s: *z* = 0.08, *P* = 0.94; Beggs: *z* = 0.24, *P* = 0.81) (Supplementary Fig. S3). Sensitivity analysis showed consistent results (Supplementary Fig. S4). According to GRADE, the certainty of the evidence was low (Supplementary Table S4).

Subgroup analyses are reported in Supplementary Fig. S5. Results varied similarly to UtA PI, according to HT use and clinical symptoms, with lower UtA RI in women not using HT (pooled mean: −0.07; 95% CI, −0.11 to −0.04; n = 3 studies; test of group differences vs women using HT: *P* < 0.01). The lowest UtA RI values were observed in women with severe symptoms (mean: −0.20; 95% CI, −0.22 to −0.18; n = 1 study), compared to women reporting mild symptoms (pooled mean: −0.07; 95% CI, −0.11 to −0.04; n = 3 studies).

Sensitivity analysis on studies controlling for menstrual cycle phase (follicular) reported a pooled mean UtA RI of −0.07 (95% CI, −0.11 to −0.03; *I*^2^ = 78.15%; n = 2 studies) (Supplementary Fig. S6).

#### UtA TAMX

Three studies (n = 470 women) assessed UtA TAMX, finding significantly higher values in women with fibroids (pooled mean: 18.46; 95% CI, 5.54–31.37; *I*^2^ = 93.64%), with no publication bias (Egger’s: *z* = 0.34; *P* = 0.73; Beggs: *z* = 0.00; *P* = 1.00) (Supplementary Fig. S7). According to GRADE, the certainty of the evidence was very low (Supplementary Table S4).

Subgroup analyses are reported in Supplementary Fig. S8. Larger differences were seen in pre-menopausal women (pooled mean: 28.10; 95% CI, 23.99–32.21; n = 2 studies; *P* < 0.01 compared to the single study on post-menopausal population) and those with severe symptoms (pooled mean: 27.31; 95% CI, 22.19–32.44; n = 2 studies; *P* < 0.01 compared to the single study on women with no or mild symptoms).

#### UtA PSV

Four studies (n = 655 women) reported UtA PSV in women with fibroids vs controls, finding no overall difference (pooled mean: 11.33; 95% CI, −7.30 to 29.97; *I*^2^ = 97.18%) and no publication bias (Egger’s: *z* = 0.55; *P* = 0.58; Beggs: *z* = 0.34; *P* = 0.74; Supplementary Fig. S9). However, in the leave-one-out sensitivity analysis assessing the influence of individual studies, excluding Samani *et al.* (2012) yielded a significant pooled mean difference in UtA PSV of 19.56 (95% CI, 2.96–36.15), higher in women with fibroids than in controls (Supplementary Fig. S10). According to GRADE, the certainty of the evidence was very low (Supplementary Table S4).

Subgroup analyses are reported in Supplementary Fig. S11. Higher UtA PSV was observed in pre-menopausal women (pooled mean: 26.13; 95% CI, 7.21–45.06; n = 2 studies), no difference in the single post-menopausal study (*P* < 0.01 between groups). Additionally, the single study involving women with symptomatic fibroids reported notably higher PSV (mean: 40.00; 95% CI, 32.95–47.05) compared to the three studies involving mild or asymptomatic cases (*P* < 0.01).

### Uterine stiffness

**Figure 4.**
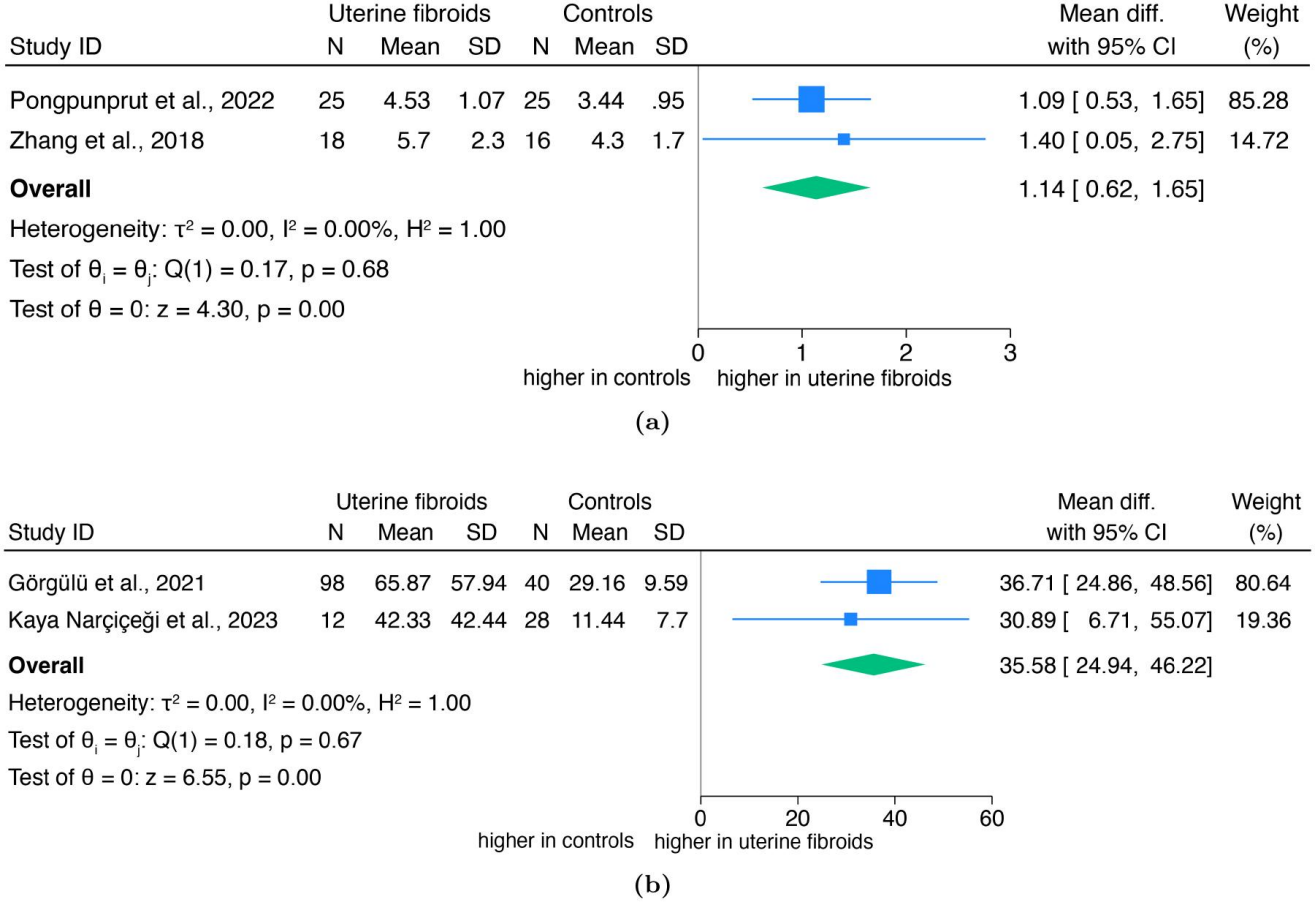
Forest plot of the meta-analysis in women with uterine fibroids vs controls on: (a) elastic modulus; (b) shear wave velocity (SWV).

A summary of the characteristics of the five included studies according to PICOS is reported in Table 1 and Supplementary Table S3. One elastography study was excluded from quantitative synthesis, as all participants had both uterine fibroids and adenomyosis (Supplementary Table S1). Two studies evaluated the elastic modulus comparing fibroids versus controls (n = 178 women), showing a significantly higher pooled mean difference of 35.58 (95% CI, 24.94–46.22), with no heterogeneity (*I*^2^ = 0%) and no small-study effect (Egger’s: *z* = −0.14; *P* = 0.89) (Fig. 4a). According to GRADE, the certainty of the evidence was very low (Supplementary Table S4).

Similarly, two studies (n = 84 women) assessing SWV found significantly higher values in fibroids compared to controls (pooled mean difference: 1.14; 95% CI, 0.62–1.65; *I*^2^ = 0%), with no publication bias (Egger’s: *z* = 0.42; *P* = 0.68) (Fig. 4b). According to GRADE, the certainty of the evidence was very low (Supplementary Table S4). Due to limited available data, subgroup analyses by menopausal status, HT, or clinical symptoms were not feasible.

Applying the vote-counting synthesis method to the four studies reporting on uterine stiffness, all showed a consistent effect toward higher stiffness in fibroids compared with controls (4/4 studies; observed proportion 100.0%, 95% CI, 51.0–100.0%; two-sided *P* = 0.13). Notably, two of these studies evaluated elasticity in women with highly symptomatic fibroids—one using elastic modulus and one using SWV—both performed during the proliferative phase of the cycle, and consistently reported higher stiffness in symptomatic fibroids (2/2 studies; observed proportion 100.0%, 95% CI 34.2–100.0%; two-sided *P* = 0.5).

### Uterine contractility

Two studies evaluating uterine contractility could not be combined quantitatively (reasons detailed in Supplementary Table S1).

Kido *et al.* (2014) assessed uterine peristalsis using 3T MRI in 20 symptomatic women with fibroids compared to 20 healthy controls. All assessments were made in peri-ovulatory phase. Peristalsis was significantly reduced in symptomatic fibroid patients, both in terms of presence and frequency of contractions, with no relationship to fibroid characteristics (localization, number, and size). The direction of peristalsis was similar in cases and controls (predominantly cervix-to-fundus).

Orisaka *et al.* (2007), using 1.5T MRI, found abnormal peristaltic patterns (e.g. direction of contractile waves) in 19 women with fibroids during menstruation and mid-luteal phase compared to controls. However, the assessment of only three controls limited quantitative comparisons.

## Discussion

### Main findings

This meta-analysis demonstrates that women with uterine fibroids, a morphological myometrial pathology, exhibit changes across multiple determinants of uterine functionality, including vascularization, stiffness, and contractility.

Despite high heterogeneity, pooled mean differences showed lower UtA PI, slightly lower UtA RI, and higher UtA TAMX in women with uterine fibroids compared to controls. Higher UtA PSV was also consistently observed in women with fibroids across all studies, with the exception of one (Samani *et al.*, 2012). Subgroup analyses found greater uterine perfusion differences among pre-menopausal women and those not using hormonal medications. Analyses limited to the follicular phase yielded consistent results and resolved heterogeneity, though data were limited. A trend related to clinical presentation was observed, with more pronounced perfusion differences compared to controls in women with symptomatic fibroids.

Though elastography data were limited, pooled results consistently showed higher elastic modulus and SWV, with no heterogeneity. Limited evidence on contractility suggests reduced peristalsis and abnormal contraction dynamics in symptomatic fibroids.

Finally, it is worth highlighting that we failed to identify any study considering concomitantly all these aspects.

### Interpretation

The finding of increased uterine perfusion and stiffness, and possibly impaired contractility, in non-pregnant women with uterine fibroids compared to controls without myometrial pathology supports the rationale for investigating uterine morphological-to-functional changes in a unified pathophysiological manner. The specific observed findings related to uterine fibroids may be explained by a range of structural, hormonal, and functional factors.

From a hemodynamic perspective, the lower UtA PI and RI observed in fibroids compared to controls likely reflect reduced uterine vascular resistance, whereas the higher UtA TAMX and PSV may indicate increased blood flow velocity. Together, these Doppler findings point to enhanced uterine vascularization, in line with the angiogenic activity that characterizes fibroid tissue, and suggest a hemodynamic profile driven by the elevated metabolic demands of fibroids (Tal and Segars, 2014). Traditionally, increased uterine perfusion observed in women with fibroids has been attributed to the overall enlargement of the uterus and the size and location of the fibroids themselves (Xue *et al.*, 2024). However, more comprehensive evidence suggests a more complex interplay between uterine perfusion and clinical presentation in women with uterine fibroids (Ciarmela *et al.*, 2022). In this view, enhanced vascularization may sustain fibroid growth and contribute to symptom development, with perfusion patterns likely modulated by hormonal regulation. Consistently, our subgroup analyses revealed that perfusion changes were more pronounced in pre-menopausal women not receiving HT and in those with symptomatic fibroids compared to controls. These observations are supported by studies indicating altered uterine perfusion in women experiencing severe dysmenorrhea, where ischemia–reperfusion injury has been proposed as a potential mechanism, reminiscent of myocardial stress injury (Iacovides *et al.*, 2015; Cockrum *et al.*, 2024). Of note, hemodynamic dysfunction in fibroid patients may extend beyond the uterus, with emerging evidence suggesting a systemic vascular component potentially driven by estrogen-mediated endothelial dysfunction (Kirschen *et al.*, 2021).

From a mechanical perspective, our synthesis indicates that uterine stiffness is consistently increased in fibroids compared with controls, whether assessed by elastic modulus or SWV, and irrespective of the measurement method, as shown in the vote-counting analysis. Although the limited number of available studies prevents definitive conclusions, histological and single-cell evidence clearly demonstrate that fibroid tissue accumulates extracellular matrix (ECM), resulting in increased stiffness and activation of profibrotic signaling pathways (Boldu-Fernández *et al.*, 2025). Tissue stiffening in fibroids leads to enhanced activation of mechanosensitive pathways, which is coupled with altered responsiveness to estrogen and progesterone signaling (Purdy *et al.*, 2020). This hormone–mechanical crosstalk is increasingly recognized as a determinant of cell fate in hormone-sensitive tissues, and its disruption may contribute to the initiation and progression of myometrial pathologies, including fibroids (Northey and Weaver, 2022). The convergence of mechanical and hormonal factors with profibrotic and inflammatory signaling also provides a plausible link between myometrial remodeling and altered uterine functionality. In particular, these processes may underlie the abnormal peristalsis and impaired contractile patterns observed in women with fibroids, although evidence remains limited. Under physiological conditions, uterine contractility displays cyclical variations across the menstrual cycle, regulated by estradiol released from the dominant follicle and progesterone secreted by the corpus luteum: cervix-to-fundus contractions prevail during the peri-ovulatory phase, facilitating sperm transport and implantation, whereas fundus-to-cervix contractions dominate during menses to aid menstrual shedding (Salmeri *et al.*, 2024). Altered peristaltic activity is increasingly recognized as a key feature of uterine disorders, with the junctional zone endometrium (JZE) emerging as a central anatomical landmark under hormonal control (Tanos *et al.*, 2019). In this context, structural and functional changes in fibroids, including increased stiffness and ECM accumulation, may disrupt JZE-driven peristalsis, thereby linking myometrial remodeling to abnormal contractile patterns and fibroid-related symptoms (Tanos *et al.*, 2019). Importantly, disruption of the intrinsic functionality of the JZE leading to abnormal uterine contractility has been associated with impaired implantation (Vidal *et al.*, 2025), suggesting a potential contribution to the infertility observed in some women with fibroids that distort or interrupt the endometrial cavity. To date, one cannot also exclude that the observed changes in uterine functionality may not exclusively be a secondary event, i.e. a change secondary to the presence of fibroids. While findings specific to fibroids may not necessarily apply to all uterine disorders, hormonal regulation clearly plays a crucial role in maintaining uterine function and driving its dysfunction (Sakaguchi *et al.*, 2003). Indeed, most conditions characterized by altered myometrial morphology have been closely linked to hormonal dysregulation, particularly involving estrogens (Blake, 2007; Vercellini *et al.*, 2014). Thus, an estrogen-mediated impairment of uterine function—potentially triggered by early-life or even *in utero* hormonal exposure—could represent a common pathophysiological mechanism underlying various structural uterine disorders.

From a therapeutic perspective, HT targeting estrogen signaling through ovarian suppression, local estrogen reduction, or receptor modulation remains central to managing some proliferative uterine diseases such as uterine fibroids and adenomyosis (Deligdisch-Schor, 2020). Although current treatment efficacy has traditionally focused on reducing symptoms or lesion size, emerging evidence suggests these outcomes may also correlate with changes in uterine function. Specifically, estradiol suppression has demonstrated reductions not only in fibroid volume but also in uterine perfusion (Weeks *et al.*, 1999), consistent with our findings of largely comparable vascular variables between hormonally treated women with fibroids and controls. Hormonal therapies might also influence uterine contractility, as uterine peristaltic activity varies significantly throughout the menstrual cycle in response to hormonal fluctuations (Bulletti *et al.*, 2000; de Ziegler *et al.*, 2001).

Therefore, to fully elucidate therapeutic mechanisms and evaluate treatment outcomes accurately, a multidimensional assessment of uterine function is essential (Munro *et al.*, 2014). In this context, early and targeted hormonal management could be critical not only for preserving healthy uterine morphology but also for maintaining or restoring optimal functional dynamics. It may also help to discern between fibroids that do and do not cause symptoms. Fibroids are extremely prevalent, and, in clinical practice, it can be difficult to disentangle whether they display detrimental effects. This situation is of utmost clinical relevance for infertility but may be important also when the reported symptoms are pain or abnormal uterine bleeding.

### Strengths and limitations

The primary strength of this meta-analysis is that it represents the first integrated, multidimensional evaluation of uterine function by simultaneously comparing three key domains—vascularization, elastography, and contractility—in women with uterine fibroids versus controls with normal myometrial morphology. The selection of these functional domains is justified by robust evidence supporting their clinical relevance. Transvaginal color Doppler ultrasound has historically provided accurate assessments of the functional state of reproductive organs, including both the uterus and ovaries (Kupesic and Kurjak, 1997). Elastography, particularly SWV techniques, offers precise quantification of tissue stiffness (Barrett *et al.*, 2024) and has been proposed by experts as a potential screening tool for early detection and intervention in fibroid management (Ali *et al.*, 2023). Conversely, the assessment of uterine contractility remains challenging due to the inherent complexity of non-invasively measuring excitation–contraction coupling in myometrial tissue (Aguilar and Mitchell, 2010); thus, conclusions drawn regarding contractility must be considered preliminary. An additional strength of this meta-analysis is the careful consideration of clinical confounding factors in the vascularization domain, such as HT, menopausal status, and symptom severity, each of which significantly influenced the observed differences.

However, several limitations must be acknowledged when interpreting our findings. The main limitation is the limited availability of evidence, especially regarding elastography and contractility, leading to low or very low certainty of the evidence. Additionally, differences in measurement methodologies across studies could introduce biases or systematic errors (Supplementary Table S2). Beyond methodological variability, heterogeneity may also have been influenced by confounding due to differences in study populations. The only study that did not report age distribution (Samani *et al.*, 2012) was also the one showing an opposite direction of effect in UtA PSV, suggesting that unbalanced age may have acted as a confounder. Although accounted for in the leave-one-out sensitivity analysis, residual confounding cannot be fully excluded. Nonetheless, despite significant heterogeneity, the consistent direction of observed effects supports the validity of our conclusions, which focused primarily on theoretical interpretation rather than providing precise pooled estimates.

## Conclusions

Since the publication of the Morphological Uterus Sonographic Assessment (MUSA) criteria in 2015 (Van den Bosch *et al.*, 2015), ultrasound-based characterization of uterine morphology has markedly advanced. However, morphological assessment alone cannot fully capture the complexity of uterine function. Uterine fibroids, with their distinctive and easily recognizable ultrasound features, offer an ideal model for investigating how structural changes in the myometrium relate to functional alterations. In this study, we observed that fibroids are associated with increased uterine perfusion, elevated tissue stiffness, and dysregulated contractility, supporting the concept that morphological abnormalities are paralleled by functional disruptions.

These functional changes may have direct clinical implications, as altered vascularization, stiffness, and contractility likely contribute to impaired fertility, abnormal uterine bleeding, pelvic pain, and other fibroid-related symptoms (Yang *et al.*, 2022). In this context, the JZE emerges as a central anatomical landmark under hormonal control, whose structural and functional alterations may underlie both symptoms and reduced fertility—particularly in cases of endometrial cavity distortion caused by submucosal or large intramural fibroids, where surgical removal may be beneficial (Ferrari *et al.*, 2024). Recognizing these mechanisms underscores the need for functional assessment in routine clinical evaluation and may help refine treatment strategies and personalize symptom management.

Looking beyond fibroids and considering other uterine disorders, we propose that future research should adopt an integrated approach to multidimensional functional assessment, aiming to enable earlier detection and improve the accuracy of treatment response evaluation. Nonetheless, important gaps remain. Current evidence is limited by small sample sizes, heterogeneous methodologies, and a lack of standardized noninvasive measures of uterine function. Therefore, future studies should prioritize the development and validation of reproducible techniques, including imaging-based and *in vivo* electrophysiological assessments, ideally integrating multidimensional evaluation of uterine functionality. A systems engineering framework may be particularly helpful in structuring this research agenda, supporting a more comprehensive understanding of vascular regulation, tissue remodeling, and inflammation-related changes in fibroids, together with deeper insights into the contractile apparatus and Ca^2+^ sensitivity regulation. Since these mechanisms are finely tuned by cycle-dependent hormonal fluctuations and are further influenced by HTs and individual reproductive history, they should be mandatorily taken into account when assessing uterine function in both research and clinical practice.

As the biomedical field moves continuously toward more sophisticated integration of data—through clinical-radiologic artificial intelligence (Rajpurkar and Lungren, 2023) or systems biology approaches such as radiomics and multi-omics (Gomes and Ashley, 2023)—there is a pressing need to bridge structural imaging of the uterus with functional quantification. Only through this lens can we begin to truly understand the physiological relevance of this historically underexplored organ.

## Supplementary Material

hoaf060_Supplementary_Data

## Data Availability

Data will be made available to the editors for review or query upon request.
