# Supplementary material for: Decoding uterine (dys)function in fibroids through multimodal assessment of functional determinants: a systematic review and meta-analysis
Source: Hum Reprod Open. 2025 Sep 18;2025(4):hoaf060. doi: 10.1093/hropen/hoaf060 (PMC12527344; doi:10.1093/hropen/hoaf060)
Supplement: hoaf060_Supplementary_Data [file hoaf060_supplementary_data.zip › HROPEN-25-0232.R2 Supplementary Figures.pdf]

Decoding uterine (dys)function in fibroids through multimodal assessment of functional determinants: a systematic review and meta-analysis

Supplementary Figures

List of Supplementary Figures

Supplementary Figure S1 Leave-one-out meta-analysis of uterine artery pulsatility index (UtA PI) in women with fibroids vs. controls. 2
Supplementary Figure S2 Sensitivity analysis of uterine artery pulsatility index (UtA PI) in women with fibroids vs. controls in the follicular phase of the menstrual cycle. 3
Supplementary Figure S3 Forest plot of the meta-analysis of uterine artery resistance index (UtA RI) in women with uterine fibroids vs. controls. 4
Supplementary Figure S4 Leave-one-out meta-analysis of uterine artery resistance index (UtA RI) in women with fibroids vs. controls. 5
Supplementary Figure S5 Sub-group analysis of uterine artery resistance index (UtA RI) in women with uterine fibroids vs. controls according to hormonal treatment and clinical presentation. 6
Supplementary Figure S6 Sensitivity analysis of uterine artery resistance index (UtA RI) in women with fibroids vs. controls in the follicular phase of the menstrual cycle. 7
Supplementary Figure S7 Forest plot of the meta-analysis on uterine artery time-averaged maximum velocity (UtA TAMX) in women with uterine fibroids vs. controls. 8
Supplementary Figure S8 Sub-group analysis of uterine artery time-averaged maximum velocity (UtA TAMX) in women with uterine fibroids vs. controls according to menopausal status and clinical presentation. 9
Supplementary Figure S9 Forest plot of the meta-analysis on UtA PSV in women with uterine fibroids vs. controls. 10
Supplementary Figure S10 Leave-one-out meta-analysis of uterine artery peak systolic velocity (UtA PSV) in women with fibroids vs. controls. 11
Supplementary Figure S11 Sub-group analysis on uterine artery peak systolic velocity (UtA PSV) in women with uterine fibroids vs. controls according to menopausal status, hormonal treatment and clinical presentation. 12

Supplementary Figure S1: Leave-one-out meta-analysis of uterine artery pulsatility index (UtA PI) in women with fibroids vs. controls.

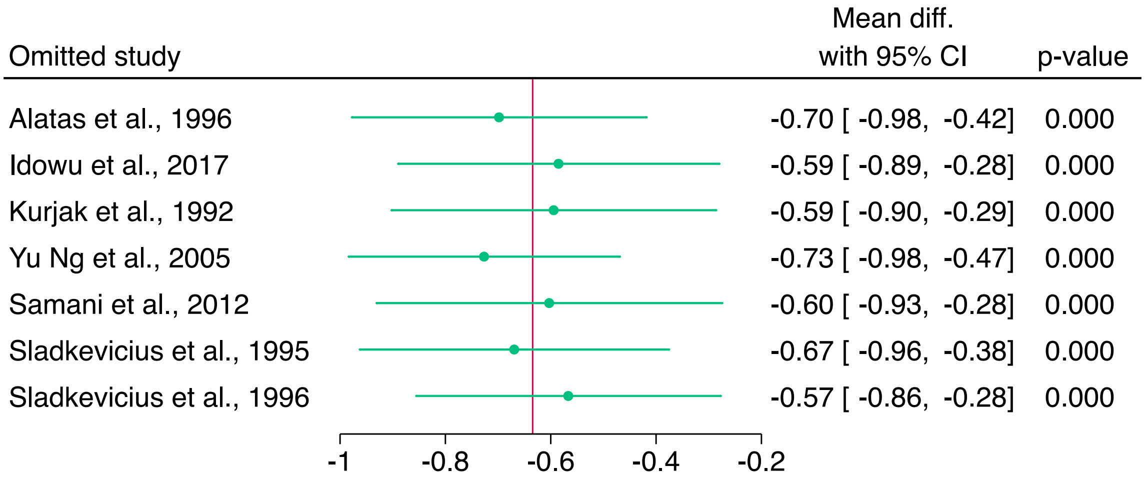

Abbreviations: CI = confidence interval.

Supplementary Figure S2: Sensitivity analysis of uterine artery pulsatility index (UtA PI) in women with fibroids vs. controls in the follicular phase of the menstrual cycle.

	Uterine fibroids			Controls			Mean diff.	Weight
Study ID	N	Mean	SD	N	Mean	SD	with 95% CI	(%)
Alatas et al., 1996	100	1.68	0.47	60	1.97	0.49	-0.29 [-0.44, -0.14]	52.55
Kurjak et al., 1992	71	1.65	0.49	60	2.52	0.87	-0.87 [-1.11, -0.63]	47.45
**Overall**							-0.57 [-0.97, -0.16]	

Heterogeneity: $\tau^2 = 0.07$, $I^2 = 87.64\%$, $H^2 = 8.09$

Test of $\theta_i = \theta_j$: Q(1) = 16.22, p = 0.00

Test of $\theta = 0$: z = -2.76, p = 0.01

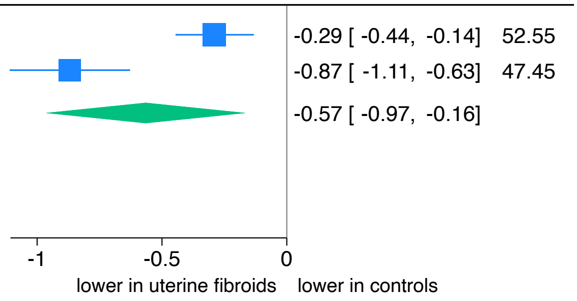

Abbreviations: SD = standard deviation; CI = confidence interval.

Supplementary Figure S3: Forest plot of the meta-analysis of uterine artery resistance index (UtA RI) in women with uterine fibroids vs. controls.

Study ID	Uterine fibroids N	Mean	SD	Controls N	Mean	SD	Mean diff. with 95% CI	Weight (%)
Alatas et al., 1996	100	0.78	0.08	60	0.82	0.06	-0.04 [-0.06, -0.02]	21.20
Idowu et al., 2017	140	0.70	0.10	140	0.90	0.03	-0.20 [-0.22, -0.18]	21.49
Kurjak et al., 1992	71	0.74	0.09	60	0.84	0.09	-0.10 [-0.13, -0.07]	20.75
Yu Ng et al., 2005	50	0.82	0.09	50	0.82	0.07	0.00 [-0.03, 0.03]	20.70
Samani et al., 2012	50	0.74	0.19	50	0.85	0.23	-0.11 [-0.19, -0.03]	15.87
**Overall**							-0.09 [-0.15, -0.03]	

Heterogeneity: $\tau^2 = 0.00$, $I^2 = 95.86\%$, $H^2 = 24.16$

Test of $\theta_i = \theta_j$: Q(4) = 183.84, p = 0.00

Test of $\theta = 0$: z = -2.79, p = 0.01

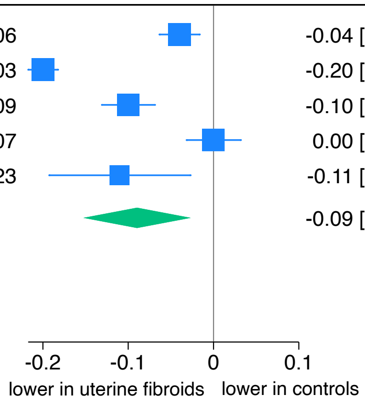

Abbreviations: SD = standard deviation; CI = confidence interval.

Supplementary Figure S4: Leave-one-out meta-analysis of uterine artery resistance index (UtA RI) in women with fibroids vs. controls.

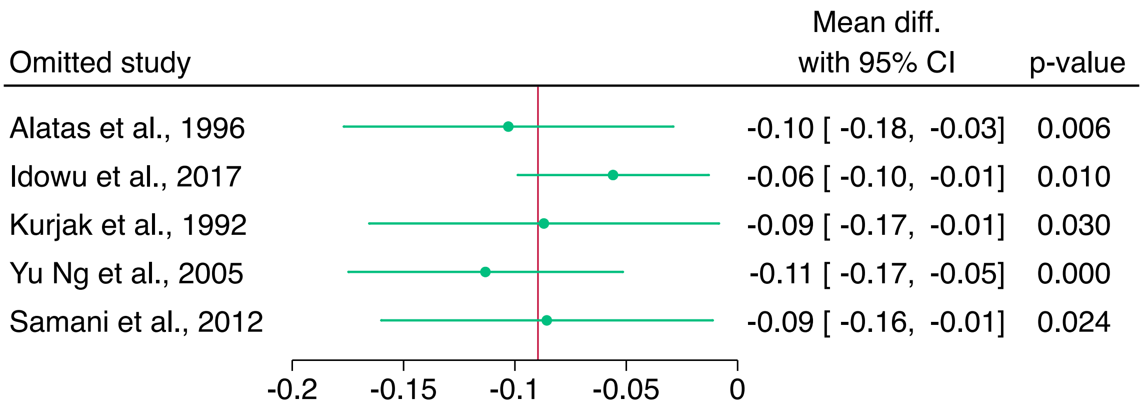

Abbreviations: CI = confidence interval.

Supplementary Figure S5: Sub-group analysis of uterine artery resistance index (UtA RI) in women with uterine fibroids vs. controls according to hormonal treatment and clinical presentation.

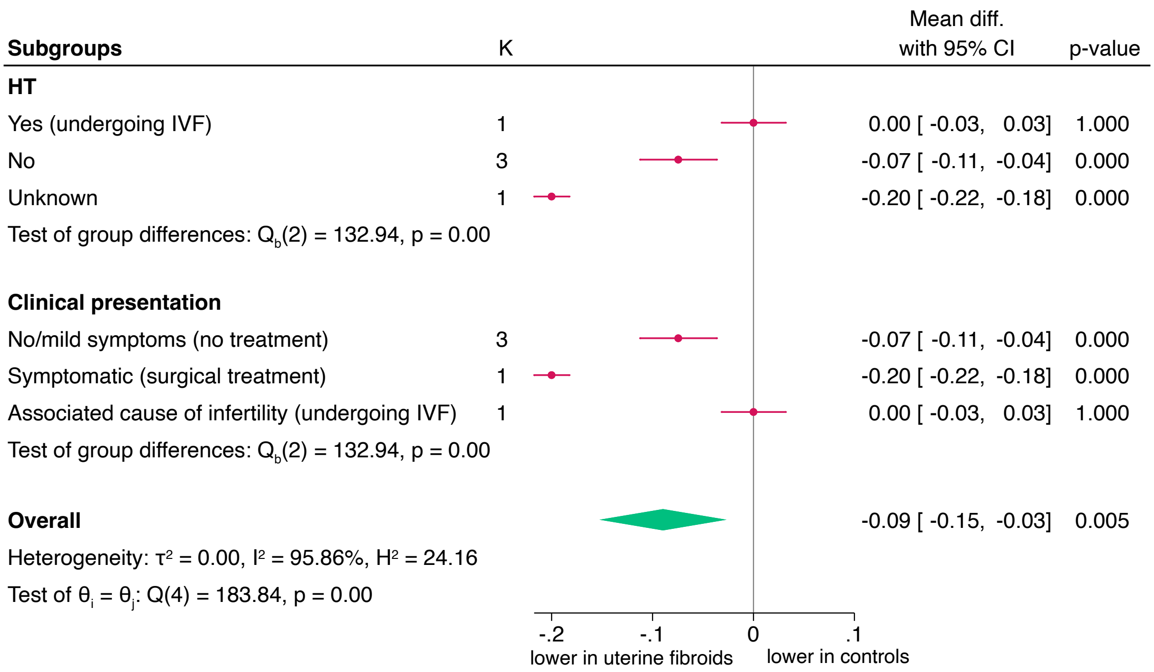

Abbreviations: CI = confidence interval; HT = hormonal treatment; IVF = in vitro fertilization.

Supplementary Figure S6: Sensitivity analysis of uterine artery resistance index (UtA RI) in women with fibroids vs. controls in the follicular phase of the menstrual cycle.

	Uterine fibroids			Controls			Mean diff.	Weight
Study ID	N	Mean	SD	N	Mean	SD	with 95% CI	(%)
Alatas et al., 1996	100	0.78	0.08	60	0.82	0.06	-0.04 [-0.06, -0.02]	52.96
Kurjak et al., 1992	71	0.74	0.09	60	0.84	0.09	-0.10 [-0.13, -0.07]	47.04
**Overall**							-0.07 [-0.11, -0.03]	

Heterogeneity: $\tau^2 = 0.00$, $I^2 = 78.15\%$, $H^2 = 4.58$

Test of $\theta_i = \theta_j$: $Q(1) = 9.19$, $p = 0.00$

Test of $\theta = 0$: $z = -3.23$, $p = 0.00$

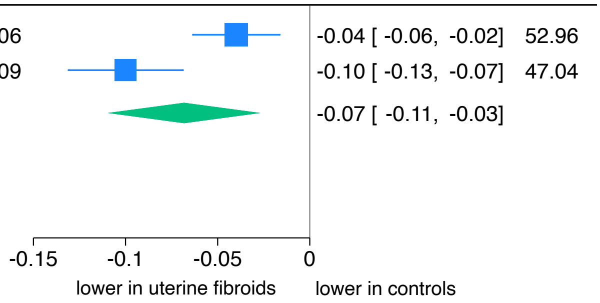

Abbreviations: SD = standard deviation; CI = confidence interval.

Supplementary Figure S7: Forest plot of the meta-analysis on uterine artery time-averaged maximum velocity (UtA TAMX) in women with uterine fibroids vs. controls.

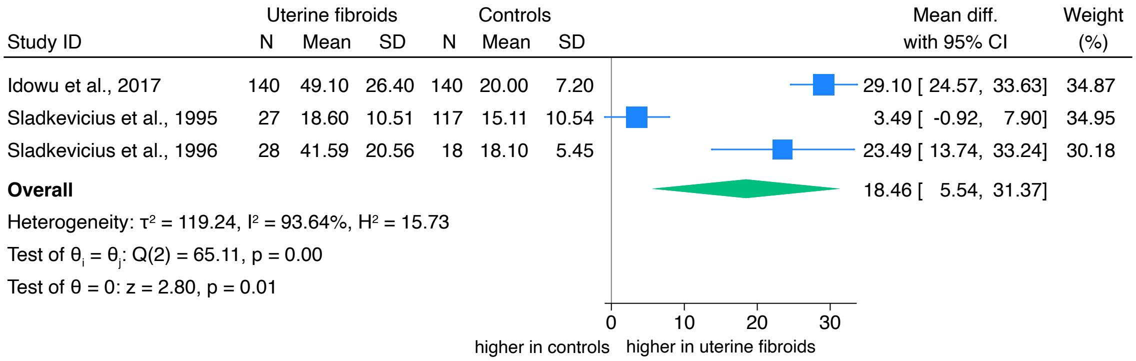

Abbreviations: SD = standard deviation; CI = confidence interval.

Supplementary Figure S8: Sub-group analysis of uterine artery time-averaged maximum velocity (UtA TAMX) in women with uterine fibroids vs. controls according to menopausal status and clinical presentation.

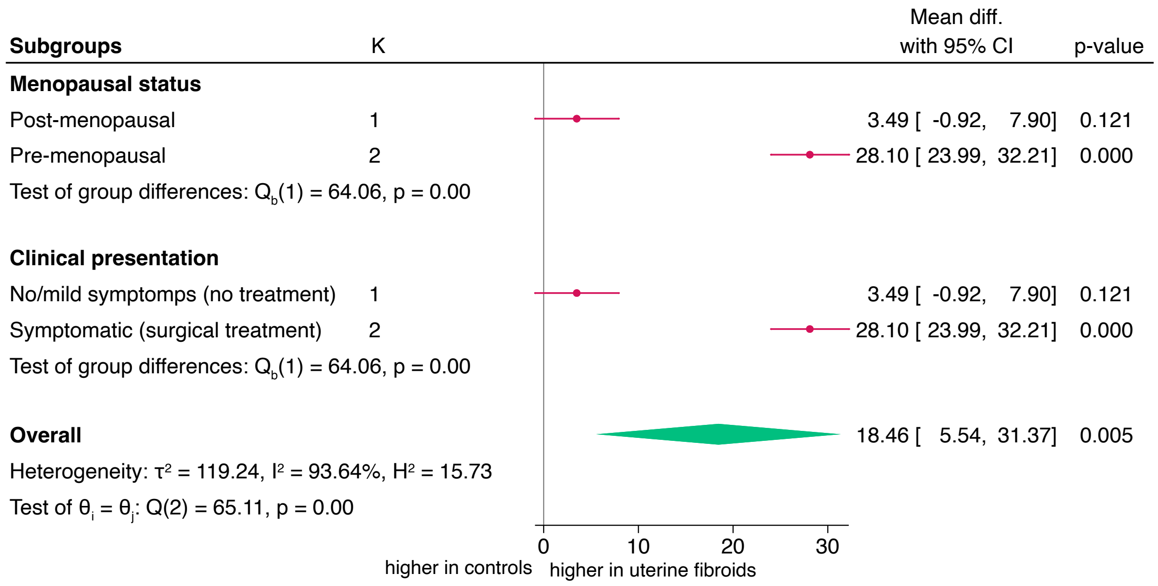

Abbreviations: CI = confidence interval.

Supplementary Figure S9: Forest plot of the meta-analysis on UtA PSV in women with uterine fibroids vs. controls.

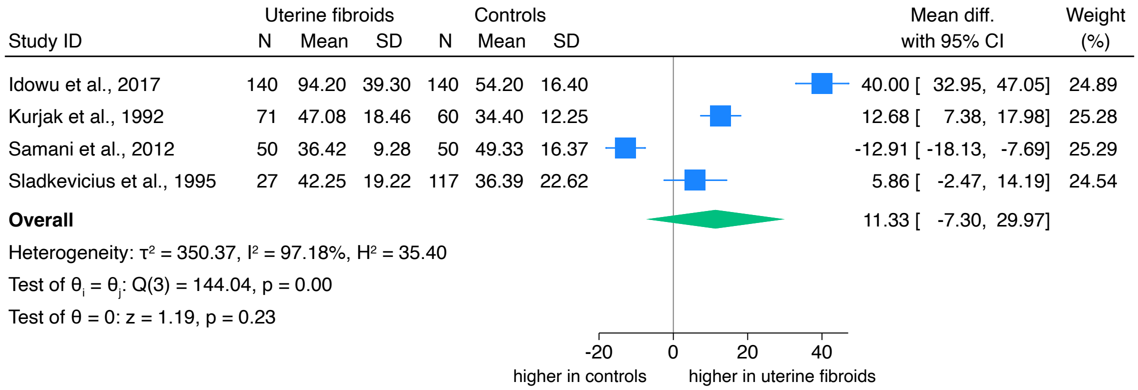

Abbreviations: UtA PSV = uterine artery peak systolic velocity; SD = standard deviation; CI = confidence interval.

Supplementary Figure S10: Leave-one-out meta-analysis of uterine artery peak systolic velocity (UtA PSV) in women with fibroids vs. controls.

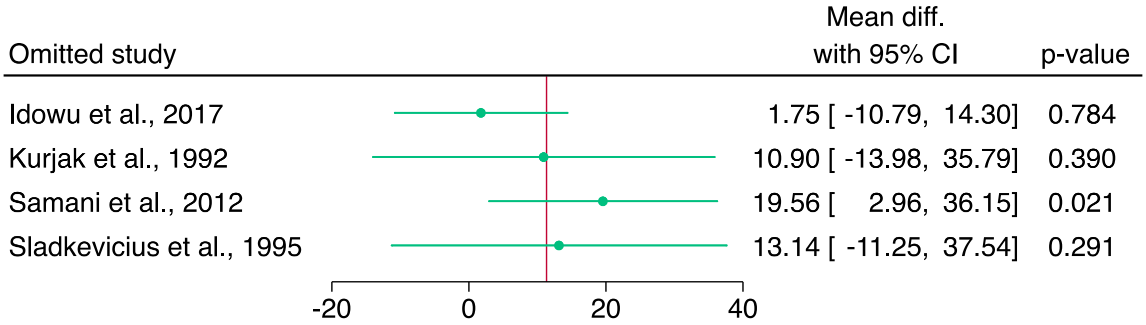

Abbreviations: CI = confidence interval.

Supplementary Figure S11: Sub-group analysis on uterine artery peak systolic velocity (UtA PSV) in women with uterine fibroids vs. controls according to menopausal status, hormonal treatment and clinical presentation.

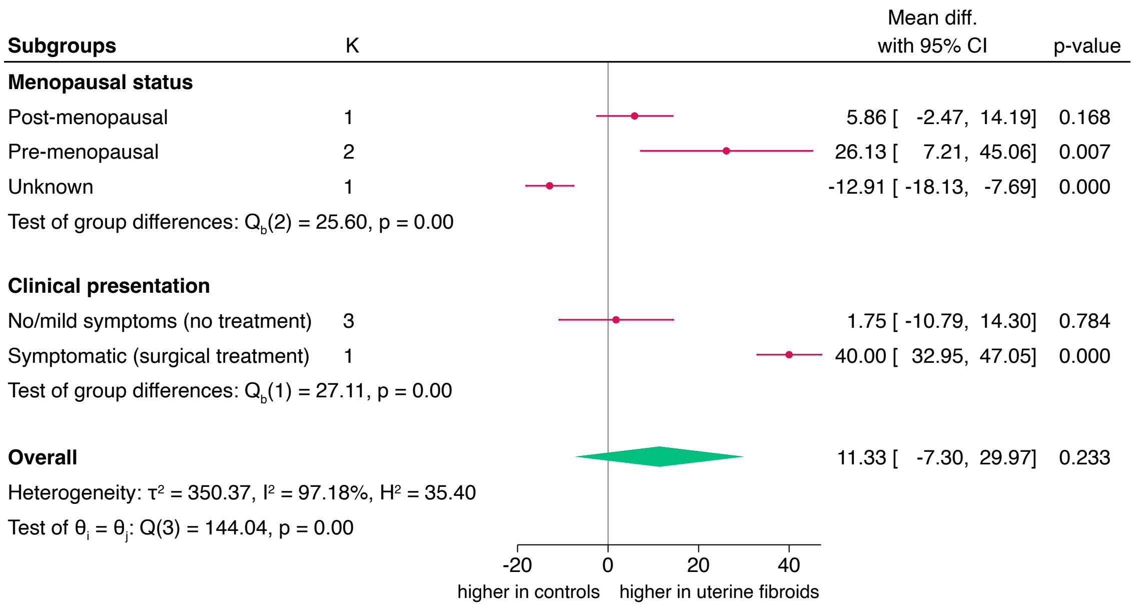

Abbreviations: CI = confidence interval.
