# Supplementary material for: Decoding uterine (dys)function in fibroids through multimodal assessment of functional determinants: a systematic review and meta-analysis
Source: Hum Reprod Open. 2025 Sep 18;2025(4):hoaf060. doi: 10.1093/hropen/hoaf060 (PMC12527344; doi:10.1093/hropen/hoaf060)
Supplement: hoaf060_Supplementary_Data [file hoaf060_supplementary_data.zip › Supplementary Files.docx]

**Supplementary File S1.** Search strategy.

Final search date: December 20^th^, 2024

**PubMed**

| **Search** | **Query** | **Items found** |
| --- | --- | --- |
| **#4** | #1 AND #2 AND #3 | **606** |
| **#3** | (measur*[tiab] OR quant*[tiab] OR ultrasound[tiab] OR US[tiab] OR TVUS[tiab] OR sonogr*[tiab] OR MRI[tiab] OR resonance[tiab] OR electroph*[tiab] OR elastog*[tiab] OR doppler[tiab] OR "strain imaging"[tiab] OR "3D ultrasound"[tiab] OR "imaging"[tiab]) | **7,961,683** |
| **#2** | (uterine fibroid[MeSH Terms] OR myoma[MeSH Terms] OR leiomyoma[MeSH Terms] OR fibromyoma[MeSH Terms] OR myoma*[tiab] OR fibroid*[tiab] OR leiomyoma*[tiab]) | **37,232** |
| **#1** | ("uterine, contraction"[MeSH Terms] OR "pressure"[MeSH Terms] OR contract*[tiab] OR peristal*[tiab] OR wave*[tiab] OR pressure[tiab] OR pulsatil*[tiab] OR “uterine, artery”[MeSH Terms] OR "uterine dynamics"[tiab] OR "uterine motility"[tiab] OR "blood flow"[tiab] OR "vascular resistance"[tiab]) | **1,993,098** |

**Embase**

| **Search** | **Query** | **Items found** |
| --- | --- | --- |
| **#4** | #1 AND #2 AND #3 | **1,615** |
| **#3** | ('measur*':ti,ab,kw OR 'quant*':ti,ab,kw OR 'ultrasound':ti,ab,kw OR 'us':ti,ab,kw OR 'tvus':ti,ab,kw OR 'sonogr*':ti,ab,kw OR 'mri':ti,ab,kw OR 'resonance':ti,ab,kw OR 'electroph*':ti,ab,kw OR 'elastog*':ti,ab,kw OR 'doppler':ti,ab,kw OR 'strain imaging':ti,ab,kw OR '3d ultrasound':ti,ab,kw OR 'imaging':ti,ab,kw) | **10,341,914** |
| **#2** | ('uterus myoma'/exp OR 'myoma'/exp OR 'leiomyoma'/exp OR 'leiomyoma'/exp OR 'myoma*':ti,ab,kw OR 'fibroid*':ti,ab,kw OR 'leiomyoma*':ti,ab,kw) | **61,221** |
| **#1** | ('uterus contraction'/exp OR 'pressure'/exp OR 'contract*':ti,ab,kw OR 'peristal*':ti,ab,kw OR 'wave*':ti,ab,kw OR 'pressure':ti,ab,kw OR 'pulsatil*':ti,ab,kw OR 'uterine artery'/exp OR 'uterine dynamics':ti,ab,kw OR 'uterine motility':ti,ab,kw OR 'blood flow':ti,ab,kw OR 'vascular resistance':ti,ab,kw) | **2,499,556** |

**Scopus**

| **Search** | **Query** | **Items found** |
| --- | --- | --- |
| **#4** | 1# AND #2 AND #3 | **2,895** |
| **#3** | (TITLE-ABS-KEY(measur* OR quant* OR ultrasound OR US OR TVUS OR sonogr* OR MRI OR resonance OR electroph* OR elastog* OR doppler OR "strain imaging" OR "3D ultrasound" OR imaging)) | **22,854,370** |
| **#2** | (TITLE-ABS-KEY("uterine fibroid" OR myoma OR leiomyoma OR fibromyoma OR myoma* OR fibroid* OR leiomyoma*)) | **52,536** |
| **#1** | (TITLE-ABS-KEY("uterine contraction" OR pressure OR contract* OR peristal* OR wave* OR pulsatil* OR "uterine artery" OR "uterine dynamics" OR "uterine motility" OR "blood flow" OR "vascular resistance")) | **8,488,556** |

**Supplementary File S2.** Data abstraction.

The following data were extracted from the original studies and organized into a table:

- First author, year of publication
- Country where the study was conducted
- Study design
- Sample size (cases and controls)
- Diagnostic modality used for identifying uterine fibroids
- Type of control group (with or without comorbidities)
- Demographic data (including age, current hormonal treatment, fertility history, and parity)
- For pre-menopausal cohorts: phase of the menstrual cycle (menstrual, mid-follicular, late follicular, early or late luteal phase), when reported
- Criteria and imaging modalities used to quantify uterine functionality (vascularization, stiffness, contractility)
- Raw data on specific measures used to quantify uterine vascularization, stiffness, contractility, in cases and controls:
  - Uterine vascularization: uterine arteries pulsatility index (PI), resistance index (RI), peak systolic velocity (PSV), time-averaged maximum velocity (TAMX);
  - Uterine stiffness, measured either by Shear Wave Elastography (SWE) and/or Strain Elastography (SE): elastic modulus, Shear Wave velocity (SWV) or strain ratio (SR) or elasticity score;
  - Uterine contractility: presence or absence of peristalsis (n, %), frequency of contractions (waves/minute), direction and pattern of contraction.
