# Supplementary material for: Decoding uterine (dys)function in fibroids through multimodal assessment of functional determinants: a systematic review and meta-analysis
Source: Hum Reprod Open. 2025 Sep 18;2025(4):hoaf060. doi: 10.1093/hropen/hoaf060 (PMC12527344; doi:10.1093/hropen/hoaf060)
Supplement: hoaf060_Supplementary_Data [file hoaf060_supplementary_data.zip › Supplementary Tables.docx]

**Supplementary Table S1.** Excluded studies and reasons for exclusion.

| **Study** | **Reason for exclusion** |
| --- | --- |
| Săsăran et al., 2022 | Excluded from the meta-analysis because all participants had concomitant uterine fibroids and adenomyosis, and no extractable data were available for pooling |
| Kido et al., 2014 | Excluded from the meta-analysis due to the lack of other studies reporting the same outcome, resulting in insufficient data for pooling |
| Orisaka et al., 2007 | Excluded from the meta-analysis as the study reported only single-case data and included three controls, preventing meaningful case-control comparisons |

**Supplementary Table S2.** Quality assessment of included studies according to Newcastle-Ottawa Scale (NOS).

| **Study** | ***Selection*** | | | | ***Comparability*** | ***Outcome*** | | | ***NOS score*** |
| --- | --- | --- | --- | --- | --- | --- | --- | --- | --- |
|  | **Q1** | **Q2** | **Q3** | **Q4** | **Q5** | **Q6** | **Q7** | **Q8** |  |
| **Uterine vascularization** | | | | | | | | | |
| *Cohort studies* | | | | | | | | | |
| Yu Ng et al., 2005 | 1 | 1 | 1 | 1 | 2 | 1 | 1 | 1 | 9 |
| *Case-control studies* | | | | | | | | | |
| Alataş et al., 1996 | 1 | 1 | 1 | 1 | 1 | 1 | 1 | 1 | 8 |
| Idowu et al., 2017 | 1 | 1 | 1 | 1 | 1 | 1 | 1 | 1 | 8 |
| Kurjak et al., 1992 | 1 | 1 | 1 | 1 | 1 | 1 | 1 | 1 | 8 |
| Samani et al., 2012 | 1 | 1 | 1 | 1 | 0 | 1 | 1 | 1 | 7 |
| Sladkevicius et al., 1995 | 1 | 1 | 1 | 1 | 1 | 1 | 1 | 1 | 8 |
| Sladkevicius et al., 1996 | 1 | 1 | 1 | 1 | 1 | 1 | 1 | 1 | 8 |
| **Uterine stiffness** | | | | | | | | | |
| *Cohort studies* | | | | | | | | | |
| Kaya Narçiçeği et al., 2023 | 1 | 1 | 1 | 1 | 1 | 1 | 1 | 1 | 8 |
| Zhang et al., 2018 | 1 | 0 | 1 | 1 | 0 | 1 | 1 | 1 | 6 |
| *Case-control studies* | | | | | | | | | |
| Görgülü et al., 2021 | 1 | 1 | 1 | 1 | 2 | 1 | 1 | 1 | 9 |
| Săsăran et al., 2022 | 1 | 1 | 1 | 1 | 0 | 1 | 1 | 1 | 7 |
| *Cross-sectional* | | | | | | | | | |
| Pongpunprut et al., 2022 | 1 | 1 | 1 | 1 | 0 | 1 | 1 | NA | 6 |
| **Uterine contractility** | | | | | | | | | |
| *Case-control studies* | | | | | | | | | |
| Kido et al., 2014 | 1 | 1 | 1 | 1 | 0 | 1 | 1 | 1 | 7 |
| Orisaka et al., 2007 | 1 | 1 | 0 | 1 | 0 | 1 | 1 | 1 | 6 |

*Cohort studies*: Q1: Representativeness of the exposed cohort; Q2: Selection of the nonexposed cohort; Q3: Ascertainment of exposure; Q4: Illustration that there was no result of interest at start of research; Q5: Comparability of cohort based on the design or analysis; Q6: Evaluation of result; Q7: Was follow up long enough for outcome to happen; Q8: Adequate of follow up of cohort.

*Case-control studies*: Q1: Adequacy of case definition; Q2: Representativeness of the cases; Q3: Selection of controls; Q4: Definition of controls; Q5: Comparability of cases and controls on the basis of the design or analysis; Q6: Ascertainment of exposure; Q7: Same method of ascertainment for cases and controls; Q8: Non-Response rate.

*Cross-sectional*: Q1: Representativeness of the sample; Q2: Sample size; Q3: Non-respondents; Q4: Ascertainment of the exposure (risk factor); Q5: Comparability of subjects in different outcome groups on the basis of design or analysis: confounding factors controlled; Q6: Assessment of outcome; Q7: Statistical test.

**Supplementary Table S3.** Imaging modalities in included studies assessing uterine functional determinants (vascularization, stiffness, and contractility) in fibroids vs. controls.

| **First author, year** | **Uterine functionality measure addressed** | **Imaging modality and setting** | **Phase of the menstrual cycle** |
| --- | --- | --- | --- |
| **Uterine vascularization (n=7 studies)^1^** | | | |
| Alataş et al., 1996 | Vascularization: UtA PI and RI | Pulsed color Doppler TVUS (PRF: NR; filter: 100 Hz; volume: NR - imaging was optimized for detecting slow-flow) | All assessments in mid-proliferative phase |
| Idowu et al., 2017 | Vascularization: UtA PI, RI, TAMX (cm/s) and PSV (cm/s) | Pulsed color Doppler TA US (PRF: NR; filter: 50-100 Hz; volume: 1mm - imaging was optimized for detecting slow-flow) | NR |
| Kurjak et al., 1992 | Vascularization: UtA PI, RI and PSV (cm/s) | Pulsed color Doppler TVUS (PRF: 2-42 kHz; filter: 100 Hz; volume: 2mm) | Assessments on day 5^th^-8^th^ of menstrual cycle |
| Yu Ng et al., 2005 | Vascularization: UtA PI and RI | Pulsed color Doppler TVUS (PRF: NR; filter: NR; volume: NR) | Assessments on the day of oocyte pick-up |
| Samani et al., 2012 | Vascularization: UtA PI, RI and PSV (cm/s) | Pulsed color Doppler TVUS (PRF: NR; filter: NR; volume: 1mm) | NR |
| Sladkevicius et al., 1995 | Vascularization: UtA PI, TAMX (cm/s) and PSV (cm/s) | Pulsed color Doppler TVUS (PRF: 5 kHz; filter: 125 Hz; volume: NR) | NR |
| Sladkevicius et al., 1996 | Vascularization: UtA PI and TAMX (cm/s) | Pulsed color Doppler TVUS (PRF: 5 kHz; filter: 125 Hz; volume: NR) | Assessments during the proliferative phase in 50% of cases; assessments on day 7^th^ after ovulation (detected by US) in controls |
| **Uterine stiffness (n=5 studies)** | | | |
| Görgülü et al., 2021 | SE, SWE  Measures: SR (mean and max)^2^, elasticity score (Tsukuba’s score 1 to 5)  elastic modulus (kPa) | SE by TVUS, SWE by TA US  Note: assessment was made with the least possible compression applied | Assessments during the first phase of the  menstrual cycle |
| Kaya Narçiçeği et al., 2023 | SWE  Measures: Elastic modulus (kPa) | SWE by TVUS  Note: assessment was made with no manual compression | NR |
| Pongpunprut et al., 2022 | SWE  Measures: SWV (m/s) | SWE by TVUS | NR |
| Săsăran et al., 2022 | SE  Measures: SR (mean and max)^3^ | SE with TVUS  Note: pressure was applied in accordance with the quality indicator of the US machine | Assessments on day 8^th^-14^th^ of menstrual cycle |
| Zhang et al., 2018 | SWE  Measures: SWV (m/s) | SWV with TVUS  Note: measurement considered invalid if the color pixels in the ROI were scant or the standard deviation was greater than 30% | NR |
| **Uterine contractility (n=2 studies)** | | | |
| Kido et al., 2014 | Presence of peristalsis, frequency (waves/3 minutes), direction of contractions (cervix-to-fundus, fundus-to-cervix, disorganized) | 3T MRI  Cine MRI: sagittal TSE and  60 serial HASTE images obtained over 3 min in the mid-sagittal plane of the uterus | All assessments in peri-ovulatory phase |
| Orisaka et al., 2007 | Presence of peristalsis (movement of the junctional zone), frequency (waves/3 minutes), direction of contractions (cervix-to-fundus, fundus-to-cervix, opposing, isthmical) | 1.5T MRI  Cine MRI: 32 SSFSE images obtained over 3-4 min in the mid-sagittal plane of the uterus | Controls assessed throughout all menstrual cycle phases; cases evaluated once (at varying phases) |

Abbreviations: HASTE = half-Fourier acquisition single shot TSE; MRI = magnetic resonance imaging; NR = not reported; PI = pulsatility index; PRF = Pulse Repetition Frequency; PSV = Peak Systolic Velocity; ROI = region of interest; RI = resistance index; SE = Strain Elastography; SR = strain ratio; SSFSE = serial T2-weighted single-shot fast spin-echo; SWE = Shear Wave Elastography; SWV = Shear Wave Velocity; TA US = transabdominal ultrasound; TAMX = Time-Averaged Maximum Velocity; TSE = turbo spin-echo; TVUS = transvaginal ultrasound; UtA = Uterine arteries.

Notes:

^1^All the included studies reported that assessment was made at the level of the internal cervical os.

^2^Data reported only on uterine fibroids and adenomyosis. No data on controls available for comparisons.

^3^Not sufficient number of studies to compute a pooled estimate on SR (mean and max).

**Supplementary Table S4.** GRADE evidence profile for quality assessment of included studies.

|  | **GRADE assessment** | | | | | **Summary of findings** |
| --- | --- | --- | --- | --- | --- | --- |
| Outcome | Limitations | Inconsistency | Indirectness | Imprecision | Publication bias |  |
| **Uterine vascularization** | | | | | | |
| UtA PI | Low-Moderate limitations:  Seven studies included.  Studies at low or unclear risk of bias.  All the studies used the same imaging modality (gold standard) to assess the outcome. | High inconsistency:  Minimal overlap of CIs.  High heterogeneity: I2=91.98% | Low indirectness:  All studies performed examination at the level of the internal cervical os and with optimized Doppler settings.  Large difference in mean age of included studies, but almost all with populations in pre-menopausal status (with subgroup analyses to address confounding effect).  Differences in HT and clinical presentation (confounding factors) addressed by subgroup analyses.  Mostly undefined phase of the menstrual cycle (with subgroup analyses during the follicular phase to address confounding effect). | Low imprecision:  The 95% CIs exclude no effect.  All the studies’ estimates are on the same side of the decision-making threshold. Only 2 studies’ 95% CIs include the decision-making threshold, exceeding the other side only by a small margin. | Low publication bias:  Most of the included studies encompass a small population.  No significant publication bias according to Egger’s Test (p=0.97) and Begg’s Test (p=0.76). | Low |
| UtA RI | Low-Moderate limitations:  Only 5 studies included.  Studies at low or unclear risk of bias.  All the studies used the same imaging modality (gold standard) to assess the outcome. | High inconsistency:  Minimal overlap of CIs.  High heterogeneity: I2=95.86% | Low indirectness:  All studies performed examination at the level of the internal cervical os and with optimized Doppler settings.  Large difference in mean age of included studies, but all populations in pre-menopausal status.  Differences in HT and clinical presentation (confounding factor) addressed by subgroup analyses.  Mostly undefined phase of the menstrual cycle (with subgroup analyses during the follicular phase to address confounding effect). | Low imprecision:  The 95% CIs exclude no effect.  All the studies’ estimates are on the same side of the decision-making threshold, except one that straddles it. Only 1 study’s 95% CIs include the decision-making threshold. | Low publication bias:  Most of the included studies encompass a small population.  No significant publication bias according to Egger’s Test (p=0.94) and Begg’s Test (p=0.81). | Low |
| UtA TAMX | Moderate Limitations:  Only 3 studies included.  Studies at low or unclear risk of bias.  All the studies used the same imaging modality (gold standard) to assess the outcome. | High inconsistency:  Minimal overlap of CIs.  High heterogeneity: I2=93.64% | Low indirectness:  All studies performed examination at the level of the internal cervical os and with optimized Doppler settings.  Large difference in mean age of included studies, but almost all with populations in pre-menopausal status (with subgroup analyses to address confounding effect).  Differences in clinical presentation (confounding factor) addressed by subgroup analyses.  Mostly undefined phase of the menstrual cycle. | Low imprecision:  The 95% CI excludes no effect.  All the studies’ estimates are on the same side of the decision-making threshold, except one that straddles it. Only 1 study’s 95% CIs include the decision-making threshold. | Low publication bias:  Most of the included studies encompass a small population.  No significant publication bias according to Egger’s Test (p=0.73) and Begg’s Test (p=1.00). | Very Low |
| UtA PSV | Moderate Limitations:  Only 4 studies included.  Studies at low or unclear risk of bias.  All the studies used the same imaging modality (gold standard) to assess the outcome. | High inconsistency:  Minimal overlap of CIs.  High heterogeneity: I2=97.18% | Low indirectness:  All studies performed examination at the level of the internal cervical os and with optimized Doppler settings.  Large difference in mean age of included studies, but almost all with populations in pre-menopausal status (with subgroup analyses to address confounding effect).  Differences in clinical presentation (confounding factor) addressed by subgroup analyses.  Mostly undefined phase of the menstrual cycle. | High imprecision:  Wide difference in studies’ estimates: one of the study is on the left side of the decision-making threshold, one is on the right side but with 95%CIs including the decision-making threshold, and half are on the right side. | Low publication bias:  Most of the included studies encompass a small population.  No significant publication bias according to Egger’s Test (p=0.58) and Begg’s Test (p=0.74). | Very Low |
| **Uterine stiffness** | | | | | | |
| Elastic modulus | Moderate-high limitations:  Only 2 studies included.  Studies at low or unclear risk of bias.  All the studies confirmed diagnosis of uterine fibroids by histopathological confirmation. | Low inconsistency:  Wide overlap of 95% CIs.  No heterogeneity: I2=0.0% | Moderate indirectness:  Populations were comparable (for age and menopausal status).  Elastography techniques were slightly different, but the setting was comparable.  The identification of the ROI and the number of ROI considered was different; in one they also didn’t consider lesions deeper than 8 cm.  Direct comparison of cases and controls was carried appropriately. | Low imprecision:  The 95% CIs of the pooled estimates are wide but excludes no effect (none of the included studies crosses the line of no effect; both studies are on the same side of decision-making threshold).  The sample size is very small in one of the two studies and moderately large in the other. | Low-moderate publication bias:  One of the included studies encompass a small population.  No significant publication bias according to Egger’s test p-value=0.89. | Very low |
| SWV | Moderate-high limitations:  Only 2 studies included.  Studies at low or unclear risk of bias.  One study at unclear or high risk of bias (not controlling for confounding factors adequately).  Eligibility criteria for one study were not clearly defined. | Low inconsistency:  Wide overlap of mean estimates and 95% CIs.  No heterogeneity: I2=0.0% | Moderate indirectness:  Elastography techniques were slightly different, but the setting was comparable.  The identification of the ROI and the number of ROI considered was different; in one they also didn’t consider lesions deeper than 3 cm while in the other they only mention some difficulties with those. | Low imprecision:  The 95% CIs of the pooled estimates excludes no effect (none of the included studies crosses the line of no effect; both studies are on the same side of decision-making threshold). | Low-moderate publication bias:  Both studies encompass a small population.  No significant publication bias according to Egger’s test p-value=0.68. | Very low |

Abbreviations: CIs = confidence intervals; PI = pulsatility index; PSV = peak systolic velocity; RI = resistance index; ROI = region of interest; TAMX = time-averaged maximum velocity; UtA = uterine artery.
